# Neurocognitive impact of different irradiation modalities for patients with grade I-II skull base meningioma: a prospective multi-arm cohort study (CANCER COG)

**DOI:** 10.1186/s13014-025-02591-1

**Published:** 2025-01-29

**Authors:** Paul Lesueur, Florence Joly, Benedicte Clarisse, Justine Lequesne, Dinu Stefan, Jacques Balosso, Marie Lange, Sebastien Thureau, Aurelie Capel, Marie Castera, Berenice Legrand, Nicolas Goliot, Jean Michel Grellard, Thomas Tessonnier, Helene Castel, Samuel Valable

**Affiliations:** 1 Department of Radiation Oncology, François Baclesse center, Caen, 14000 France; 2 ISTCT UMR 6030-CNRS, Université de Caen-Normandie, Caen, France; 3 Department of Radiation Oncology, Centre Guillaume le conquérant, Le Havre, 76600 France; 4 Department of Clinical research, François Baclesse Center, Caen, 14000 France; 5 Department of Radiation Oncology, Henri Becquerel Center, Rouen, 76000 France; 6 Heidelberg Ion Beam Therapy Center (HIT), Department of radiation Oncology, Heidelberg university Hospital, 69120 Heidelberg, Germany; 7 INSERM U1245, Université de Rouen Normandie, Rouen, 76000 France

**Keywords:** Meningioma, Skull base, Cognition, Irradiation, Proton therapy

## Abstract

**Background:**

Radiotherapy as a complement or an alternative to neurosurgery has a central role in the treatment of skull base grade I-II meningiomas. Radiotherapy techniques have improved considerably over the last two decades, becoming more effective and sparing more and more the healthy tissue surrounding the tumour. Currently, hypo-fractionated stereotactic radiotherapy (SRT) for small tumours and normo-fractionated intensity-modulated radiotherapy (IMRT) or proton-therapy (PT) for larger tumours are the most widely used techniques. It is expected a decrease of the risk of cognitive impairment with these modern techniques. However prospective data about cognitive long-term consequences of partial brain irradiation with SRT, PT, or IMRT remain very scarce to date.

**Methods:**

CANCER COG is one of the first multicentric study in the world to prospectively assess the cognitive performances of patients following different modalities of cerebral radiotherapy (stereotactic radiotherapy, proton therapy, intensity modulated radiotherapy) for the treatment of grade I-II skull base meningioma, up to at least 10 years after the end of radiotherapy. This longitudinal study includes the follow-up of 3 cohorts, including: patients treated with PRT, IMRT, and SRT. An additionally control group will be formed. The primary objective is to report long-term cognitive deterioration in each cohort until 10 years after the end of irradiation. The rate of clinical symptomatology improvement over time after irradiation, the evolution of health-related quality-of-life, anxiety/depression, fatigue, over time after irradiation, the tumoral local control after irradiation, the progression-free survival (PFS), the professional reintegration for working-age patients will also be assessed. CANCER COG aims to help clinicians to choose the best irradiation techniques with the best benefit/risk ratio. Inclusions started on september 2023.

**Trial registration:**

The study was registered on clinicaltrials.gov with the following number: NCT 06036706.

**Supplementary Information:**

The online version contains supplementary material available at 10.1186/s13014-025-02591-1.

## Background

Brain tumours represent a heterogeneous group of diseases with a variable prognosis depending on their histological type and topography. Low-grade brain tumours constitute a separate entity for which the goal of treatment is curative, and for which long patient survival is expected. Benign (grade I) and atypical (grade II) meningioma are the most frequent of them.

Radiotherapy as a complement or an alternative to neurosurgery has a central role in the treatment of these tumours. Radiotherapy techniques have improved considerably over the last two decades, becoming more effective and sparing more and more the healthy tissue surrounding the tumour. Currently, hypo-fractionated stereotactic radiotherapy for small tumours and normo-fractionated intensity-modulated radiotherapy for larger tumours or those close to high-risk structures are the most widely used techniques. To a smaller extent, because of its limited accessibility (< 100 centers worldwide), proton-therapy is a preferred technique for treating these low-grade brain tumours. Proton-therapy is a form of radiotherapy based on the use of accelerated protons, which stop at a given depth depending on their initial energy (Bragg peak). Proton-therapy thus avoids any exit dose. It is therefore dosimetrically superior to other conventional radiotherapy modalities using photons, whose substantial exit dose is only compensated by ballistic optimisation (multiple beams, fluence modulation, etc.). The sparing of healthy tissues at risk of complications is therefore maximal in the case of irradiation with a proton beam.

However, despite these technological improvements, radiotherapy may be a source of mid or long term radiation-induced complications, and having a strong impact on the patient’s personal and professional life. Among these side effect, memory and attention disorders are the complications the most feared by the radiation oncologist [1]. These cognitive problems, which can have a major effect on quality of life, can be mild to moderate, and may sometimes lead to dementia [2]. While the neurotoxicity associated with conventional brain radiotherapy (notably whole brain irradiation) is now relatively well documented [3–5], data about cognitive long-term consequences of partial brain irradiation with modern technical remain very scarce to date [6–8].

We recently showed, in a pilot study conducted among 16 patients (clinicaltrials.gov NCT02791360) who had been treated with radiotherapy for a good prognosis brain tumour at least 10 years previously, that radiotherapy appeared to accelerate brain ageing with an effect of dose, and time since completion of radiotherapy.

## Hypothesis and expected clinical outcomes

Following these observations, we herein propose the first study in France to prospectively assess the cognitive performances of patients following different modalities of cerebral radiotherapy (stereotactic radiotherapy, proton therapy, intensity modulated radiotherapy) for the treatment of low grade skull base meningioma, up to at least 10 years after the end of radiotherapy. This project is elaborated with the expertise of the National Cancer and Cognition Platform (http://www.canceretcognition.fr/), as well as of the National Clinical research Platform for Quality of Life in Oncology, both labelled by the French League against Cancer (LNC).

For the purpose of this research, we will constitute several cohorts of patients, treated either by intensity-modulated radiotherapy, stereotactic radiotherapy or proton-therapy. This will allow better understanding the cognitive and anatomical damages caused by new radiotherapy techniques and better understanding how ionising radiation (X-rays or protons) acts in the long term on brain tissue. Longitudinal follow-up will be multimodal, based on yearly multi-parametric brain MRI to assess morphological changes [9–11], in relation with dosimetric data as well as neuropsychological performances, health-related quality of life, anxiety and depression disorders, memory tasks, and socio-professional reintegration. This will notably make it possible to evaluate the relationship between dosimetric data, age at the time of treatment, region of the brain irradiated, type of radiation used, dose per fraction, neurocognitive and neuro-anatomical consequences. A Normal Tissue Control Probability (NTCP) model will be also developed. Overall, the results of this study should contribute to the improvement of treatment techniques, in particular by preserving as much as possible the significant cerebral zones (hippocampi, frontal lobe, sub-ventricular zones, etc.), and to the management of patients by proposing appropriate support measures. In the proton-therapy cohort, evaluations will make it possible to establish more precisely the place that this new irradiation strategy should occupy in the management of low grade meningioma. Importantly, we have planned to constitute a last cohort, with subjects free of any neurological disease, to make it easier the interpretation of cognitive performances over time among patients in the three brain radiation cohorts.

## Methods

CANCER COG is a multicenter prospective longitudinal study with the constitution and the follow-up of 4 cohorts, including:

- Three cohorts of patients candidate for a brain irradiation following the diagnosis of benign meningioma or atypical meningioma:
  - Cohort “IMRT”: Patients receiving normo-fractionated intensity-modulated brain irradiation with or without stereotactic positioning (IMRT, VMAT, Tomotherapy…).
  - Cohort “SRT”: Patients receiving hypo-fractionated stereotactic brain irradiation.
  - Cohort “PRT”: Patients receiving normo-fractionated proton therapy brain irradiation.
- In addition, a control group with participants free of brain disease will be constituted:
  - Control cohort: Participants without any meningioma or neurological comorbidities.

### Primary objectives and endpoints

The primary objective is to report long-term cognitive deterioration among patients with a diagnosis of skull base benign meningioma, or atypical meningioma treated by fractionated proton-therapy (PRT) or hypo-fractionated stereotactic radiotherapy (SRT) or normo-fractionated intensity-modulated radiotherapy (IMRT), 10 years after the end of irradiation.

The primary endpoint is the proportion of patients with an impairment in cognitive parameters 10 years after irradiation of skull base benign meningioma, or atypical meningioma.

Cognitive impairment is defined as a clinically significant deterioration, in comparison with baseline evaluation (before brain irradiation), in neurocognitive test scores, grouped into five cognitive domains:

- Attention/information processing speed,
- executive functioning,
- episodic memory,
- working memory,
- short term memory.

Deterioration is either defined as (i) the occurrence of cognitive impairment (a total of ≥ 5 impaired z-scores, as considered in Douw et al. 2009) among patients who were not cognitively impaired at baseline, or (ii) the worsening of cognitive impairment (≥ 2 supplementary impaired z-scores) among patients who were already cognitively impaired at baseline.

Scores will be converted to z-scores by subtracting mean and dividing by standard deviation (SD) of data of participants free of brain disease from the control group. A z-score of at least 2 SD below normative data is defined as impaired.

### Secondary objectives and endpoints

The secondary objectives are:

- at the patient level
  - To report cognitive performances over time after brain radiotherapy for the treatment of skull base grade I-II meningioma, after the end of irradiation, according to 3 modalities of radiotherapy (PRT, SRT, IMRT).
  - To evaluate, among patients according to 3 modalities of brain radiotherapy: the delay from radiotherapy start to cognitive deterioration, the rate of clinical symptomatology improvement over time after irradiation, the evolution of health-related quality-of-life, anxiety/depression, fatigue, over time after irradiation, the tumoral local control after irradiation according to rano guidelines [12] and the progression-free survival (PFS), the professional reintegration for working-age patients, the changes of cognitive functions and cognitive complaints over time after radiotherapy.
- **With regard to radiotherapy parameters**:
  - To evaluate, according to 3 modalities of brain irradiation (PRT, SRT, IMRT), the correlation between dosimetric data and cognitive deterioration onset.
  - To build tumoral control probability (TCP) and normal tissue complication probability (NTCP) on the basis of the prospective evaluations of patients.
- **With regard to brain MRI parameters**:
  - To evaluate, according to 3 modalities of brain irradiation (PRT, SRT, IMRT), alterations/modifications of grey and white matters over time after irradiation on brain MRI and their correlation with cognitive deterioration.
  - To correlate cognitive deterioration with MRI changes.
  - To constitute a biological collection to further explore potential biomarkers predictive of radio-induced toxicities.

For participants free of brain disease:

- To report cognitive performances (cognitive functions and complaints) over time using the same timetable as for patients.

### Eligibility criteria

Patients have to fulfil the following main inclusion criteria:

- Benign meningioma (grade I), or atypical meningioma (grade II).
- Histologic proven of benign meningioma, atypical meningioma or unequivocal radiological diagnosis of skull base meningioma if biopsy is recused.
- Indication of irradiation validated by a multidisciplinary meeting.
- Age > 20 years and < 65 years.
- Expected overall survival > 10 years.
- Adjuvant or exclusive irradiation is allowed.
- Signed informed consent form.
- WHO Performance status equal to 0 or 1.
- Patient affiliated to the French social health insurance.
- Patient whose neuropsychological abilities allow to follow the requirements of the protocol.

All of the following exclusion criteria must be met:

- Patient with mutation in a known predisposition gene (NF-2, SMARCE-1).
- Cerebrovascular pathology, presence of other tumors of the nervous system, congenital malformations of the nervous system, multiple sclerosis, Parkinson’s disease, organic psychosis (other than dementia), and schizophrenia.
- Other localization than skull base meningioma.
- Histology/radiological features rather different than grade I-II meningioma.
- Histologic proven grade III meningioma.
- History of epilepsy with antiepileptic drug.
- Contraindication to MRI.
- Patient with a history of brain irradiation.
- Patient with a history of cancer in the last five years (Excluding skin baso-cellular carcinoma).
- Pregnant/breastfeeding woman,
- Any geographical conditions, social and associated psychopathology that may compromise the patient’s ability to participate in the study;
- Participation in a therapeutic trial evaluating a radiotherapy schedule or a new drug or combination for less than 30 days;
- Current or past history of second neoplasm diagnosed within the last 5 years;
- Patient deprived of freedom or under guardianship.
- Hypersensibility to Gadolinium.

### Trial schedule

CANCER COG is a multicenter prospective longitudinal study with the constitution and the follow-up of 4 cohorts:

- Cohort “IMRT”: Patients receiving normo-fractionated intensity-modulated brain irradiation with or without stereotactic positioning (IMRT, VMAT, Tomotherapy…).
- Cohort “SRT”: Patients receiving hypo-fractionated stereotactic brain irradiation.
- Cohort “PRT”: Patients receiving normo-fractionated proton therapy brain irradiation.
- Control cohort: Participants without any meningioma or neurological comorbidities.

The overall duration of the project is estimated to 15 years, including 5 years of inclusion and around 10 years of participation.

### Statistical design overview

The primary endpoint is the proportion of patients with an impairment in cognitive parameters 10 years after irradiation of skull base benign meningioma, or atypical meningioma.

As there are no available data in the literature concerning the prevalence of long-term cognitive disorders after radiotherapy treatment of skull base benign meningioma, or atypical meningioma, we assume that approximately 50% of patients will present an impairment of their cognitive functions 10 years after the end of irradiation. Using a 95% confidence interval with a width of 0.2 for a small sample size, a total of 93 patients is required.

In order to document the impact of brain irradiation on cognitive performances for each of the 3 radiotherapy modalities usually used in this indication, 31 patients are required in each of the 3 cohorts (IMRT, SRT, PRT).

In addition, a control group with 31 assessable participants free of brain disease will be constituted.

In order to anticipate 15% of subjects possibly non assessable, 36 subjects per each of the 4 cohorts will be enrolled, for a total of 108 patients and 36 participants free of brain disease.

Categorical variables will be summarized using numbers and proportions. Quantitative variables will be summarized using quartiles and range (mean and standard deviation for Gaussian-like variables). The pattern of missing data will be described.

Briefly, all outcomes will be analyzed based on intention-to-treat principle. For each cohort, the Intention to Treat (ITT) population is defined as all patients included in this radiation arm, regardless of whether eligibility criteria are met and whether or not radiation therapy is used.

#### Primary objective

The proportion of patients with an impairment in cognitive parameters 10 years after irradiation of skull base grade I-II meningioma will be estimated with a 95% confidence interval in each cohort.

#### Secondary objectives

At the patient level: no comparison between arm is planned in analyses.

The Progression-Free Survival (PFS) time is defined as the time elapsed from treatment start to progression disease or death from any cause. Data of patients who did not experience event are censored at the last assessment date. PFS will be estimated by the Kaplan-Meier method. The evolution of health-related quality-of–life after irradiation will be described by mean and standard error, and compared across time by the Student t test for paired data.

With regard to radiotherapy parameters: in each cohort, the correlation between dosimetric data (quantitative data) and the occurrence of cognitive deterioration will be assessed by the non-parametric Wilcoxon Mann Whitney test.

With regard to brain MRI parameters: In each cohort, association between alterations or modifications of grey and white matters after irradiation on brain MRI and cognitive deterioration will be assessed by the exact Fisher test.

### Study sites

Two French centers are actually recruiting patients (Table 1).

**Table 1:** Study sites

Center	Town	Country	Treatment available on site
Centre François Baclesse	Caen	France	PRT &SRT &IMRT
Centre Henri Becquerel	Rouen	France	SRT &IMRT

### Study procedures

Eligible patients will be identified by the local multidisciplinary staff among patients with an indication of brain irradiation following the diagnosis of benign meningioma or atypical meningioma. The decision of radiotherapy should be made by the local multidisciplinary committee of each investigator center. Once checked all eligibility criteria, the radiation oncologist will propose the study to patients. Patients will be given study information (explanation on the study, reading and giving of the information notice). Patients will have a reflection period of the duration of their choice.

The specific exams/procedure requested in the study before inclusion (baseline visit) will be performed after consent signature and before inclusion. Inclusion will be thus performed.

The choice of the irradiation modality is at the discretion of the radiation oncologist: hypo-fractionated stereotactic radiotherapy (SRT), normo-fractionated intensity-modulated radiotherapy (IMRT), or proton-therapy (PRT) according to each center routine.

For enrolled patients, within 6 weeks before inititation of brain irradiation, the following evaluations will be performed in the investigator center. Procedures are summed up in Table 2.

**Table 2:** Study procedures

	Screening before inclusion	Baseline assessment (within 6 weeks before radiotherapy start)	During radiotherapy period (7 weeks) for 3 cohorts: - Cohort IMRT - Cohort SRT - Cohort PRT	End of radiotherapy	AFTER THE END of radiotherapy	
					6 and 12 months	Yearly up to 10 years
Study explanation and signed informed consent before any study procedures	✓					
**Patients**						
**Standard physical and neurological examination (Nano-scale)**		✓	✓ Weekly during radiotherapy		✓	✓
**Cognitive assessment** (by a trained neuropsychologist): - *Executive functions* - *Episodic memory* - *Working memory* - *Short-term memory* - *Attention/Information processing speed* - *Prospective memory (Mem-Pro* Clinic)		✓			✓	✓ **(at 3**,** 5**,** 7 and 10 years only)**
**Brain MRI** (standard follow-up):3DT1w, Diffusion (b = 0 and b = 1000 s.mm-2, 30 directions), Perfusion (Dynamic Susceptibility Contrast, DSC-MRI), 3DT1 Gadolinium, 3D Fluid Attenuated Inversion Recovery (FLAIR), Angiographic “time of flight” sequence (TOF)		✓			✓	✓
**Cognitive complaints**,** anxiety and depression assessment** *Fact-Cog* *- HADS* *MFI*		✓			✓	✓
**Quality-of-life assessment** *- EORTC QLQC30 + BN20*		✓			✓	✓
**Evaluation of the socio-professional** **reintegration**		✓			✓	✓
*At the discretion of the investigator*: Endocrinological, ophthalmological and audiometric evaluations		✓			✓ (12 months only)	✓
	*OPTIONAL*					
Blood sample banking		✓		✓	✓	✓
Participants free of brain disease						
	Baseline assessment (within 6 weeks before radiotherapy start)	6 and 12 months	Yearly up to 10 years			
**Cognitive assessment** (by a trained neuropsychologist): - *MoCA* - *Executive functions* - *Episodic memory* - *Working memory* - *Short-term memory* - *Attention/Information processing speed* - *Prospective memory (Mem-Pro* Clinic)	✓	✓	✓ **(at 3**,** 5**,** 7 and 10 years only)**			
**Cognitive complaints**,** anxiety and depression assessment** *Fact-Cog* - *HADS* - *MFI*	✓	✓	✓			
**Quality-of-life assessment** - *EORTC QLQC30*	✓	✓	✓			

#### Irradiation

Radiotherapy will be initiated within 6 weeks after inclusion and will last 7 weeks at maximum.

The localization of radiotherapy treatment will depend on the decided radiotherapy modality:

- Patients in the IMRT and SRT cohorts will thus be treated in the investigator center.
- Patients in the PRT cohort will be addressed by the investigator, as usual in case of treatment by proton-therapy, to the the site of proton therapy of Caen. The dosimetry and the pretreatment imagery will be performed by the proton therapy center.

All patients will have to complete the brain irradiation schedule in less than 45 days, from the first day of irradiation to the last day. Treatment should not be protracted over more than 45 days. Delineation should be performed on a 1-millimeter scanner and patients should be immobilized with a thermoplastic mask. Baseline MRI should be registered with the dosimetric scan to allow an accurate delineation of the target and organs at risks (OAR).

For grade I meningioma, prescription dose will be 50-54GyRBE (1,8–2 Gy per fraction) for IMRT and PRT cohorts or 25 Gy (5 Gy per fraction) (D2% between 125 and 140%) for SRT cohort.

For grade II meningioma, prescription dose will be 54-60GyRBE (1,8–2 Gy per fraction) for IMRT and PRT cohorts. SRT is not allowed.

Details about OAR delineations and dosimetric dose constraints are given in the Supplementary material n°1.

#### Follow up after irradiation

Evaluations will be realized **at 6 months**,** 1 year and every year until 10 years after the end of radiotherapy (**Table 2). Cognitive assessment will be performed only at 6 months, 1 year, 3 years, 5 years, 7 years and 10 years.

#### Patient withdrawal

The participation to the study will be interrupted at any time under the following circumstances:

- Patient request (data already collected during the study will be stored and used unless the patient expresses his/her opposition, withdrawal of consent for further treatment).
- Patient may stop to participate to the study at any time, on his/her request, without any requirement to justify the study withdrawal. The withdrawal may be given orally or in writing. However, the investigator is responsible to take note of reason for study withdrawal, without question the patient’s wish, and has to document it, as well as the date of withdrawal.
- Concomitant disease or other reason requiring the discontinuation of study participation.
- Investigator’s request (with detailed documentation of reasoning).
- Non-compliance of patient.
- patient lost to follow-up.
- Trial termination by the sponsor.
- Death.

Any patient who prematurely withdraws from the study treatment only will continue to be followed, unless she withdraws from the study.

## Discussion

Given the excellent prognosis of skull base meningioma, every effort should be done to avoid radiation induced long-term toxicities, particularly, neuro cognitive impairment. Modern technical of irradiation are widely used in this indication, and lead to a better protection of healthy tissue.

However the clinical proof level of these technical (IMRT, PRT, SRT), concerning any cognitive benefit, remains poor. Randomized trials are difficult to set up due to issues such as access to proton-therapy.facilities. CANCER COG results will be crucial to help radiation oncologists to choose the most appropriate irradiation technical.

First inclusions started on August 2023. Final results are expected between 2030 and 2035.

## Electronic supplementary material

Below is the link to the electronic supplementary material.

Supplementary Material 1

## Data Availability

No datasets were generated or analysed during the current study.

## Abbreviations

CPP: Ethics Committee (Committee for the Protection of Persons)
DSC: Dynamic Susceptibility Contrast
ECOG: Performance statut Eastern Cooperative Oncology Group performance status
EORTC: QLQC30 + BN20 European Organisation for Research and Treatment of Cancer quality of life questionnaires
FACT-Cog: Functional Assessment of Cancer Therapy Cognitive Scale
FLAIR: 3D Fluid Attenuated Inversion Recovery
Gy: Gray
HADS: Hospital Anxiety and Depression Scale
HVLT: Hopkins Verbal Learning Test
IMRT: Intensity-modulated radiotherapy
LNC: French League against Cancer
MFI: Multidimensional Fatigue Inventory
MoCA: Montreal Cognitive Assessment
MRI: Magnetic resonance imaging
NTCP: Normal tissue complication probability
OAR: Organs at risks
PFS: Progression-free survival
PRT: Proton-therapy
QoL: Quality of life
RANO: Response Assessment in Neuro-Oncology
RBE: Relative biological effectiveness
SRT: Stereotactic radiotherapy
TCP: Tumoral control probability
TOF: Angiographic “time of flight” sequence
VMAT: Volumetric arc therapy
WHO: World Health Organization
