# Correction: Neurocognitive impact of different irradiation modalities for patients with grade I-II skull base meningioma: a prospective multi-arm cohort study (CANCER COG)

**DOI:** 10.1186/s13014-025-02650-7

**Published:** 2025-04-29

**Authors:** Paul Lesueur, Florence Joly, Benedicte Clarisse, Justine Lequesne, Dinu Stefan, Jacques Balosso, Marie Lange, Sebastien Thureau, Aurelie Capel, Marie Castera, Berenice Legrand, Nicolas Goliot, Jean Michel Grellard, Thomas Tessonnier, Helene Castel, Samuel Valable

**Affiliations:** 1 Department of Radiation Oncology, François Baclesse center, Caen, 14000 France; 2 ISTCT UMR 6030-CNRS, Université de Caen-Normandie, Caen, France; 3 Department of Radiation Oncology, Centre Guillaume le conquérant, Le Havre, 76600 France; 4 Department of Clinical research, François Baclesse Center, Caen, 14000 France; 5 Department of Radiation Oncology, Henri Becquerel Center, Rouen, 76000 France; 6 Ion Beam Therapy Center (HIT), Department of radiation Oncology, Heidelberg university Hospital, 69120 Heidelberg, Germany; 7 INSERM U1245, Université de Rouen Normandie, Rouen, 76000 France

**Correction to**: ***Radiation Oncology*** (2025) 20:16.

10.1186/s13014-025-02591-1.

In this article, the author name Thomas Tessonnier was incorrectly written as Thomas Tessonier. The author group has been updated above and the original article has been corrected.

