# Supplementary material for: Neurocognitive impact of different irradiation modalities for patients with grade I-II skull base meningioma: a prospective multi-arm cohort study (CANCER COG)
Source: Radiat Oncol. 2025 Jan 29;20:16. doi: 10.1186/s13014-025-02591-1 (PMC11781042; doi:10.1186/s13014-025-02591-1)
Supplement: Supplementary file 1 — Supplementary Material 1 [file 13014_2025_2591_MOESM1_ESM.docx]

SUPPLEMENTARY MATERIAL N°1 : IRRADIATION PARAMETERS

All patients have to complete their irradiation schedule **in less than 45 days**, from the first day of irradiation to the last day. Treatment should not be protracted over more than 45 days. Delineation should be performed on a 1-millimeter scanner and patients should be immobilized with a thermoplastic mask. Baseline MRI should be registered with the dosimetric scan to allow an accurate delineation of the target and OAR.

***Grade I meningioma:***

- Prescription dose:
  - 50-54GyRBE (1,8-2Gy per fraction) for cohorts A and C
  - 25Gy (5Gy per fraction) (D2% entre 125 et 140%) for cohort B.
- Delineation :
  - Cohort A or C :
    - GTV=
    - CTV= GTV + 3mm with respect of natural anatomic barriers.
    - PTV= CTV + 3mm (or 2mm if stereotactic positioning)
  - Cohort B :
    - GTV= Gross tumor or tumoral bed.
    - CTV= GTV
    - PTV= CTV + 2mm

***Grade II meningioma :***

- Prescription dose (Cohort A and C):
  - - 54-60GyRBE (1,8-2Gy per fraction)
    - SRT is not allowed for grade II meningioma
- Delineation :
  - Cohorts A and C :
    - GTV= Gross tumor or tumoral bed.
    - CTV= GTV + 10mm with respect of natural anatomic barriers
    - PTV= CTV + 3mm (or 2mm if stereotactic positioning)

### Considering organ at risks:

The neuro-oncology EPTN consensus-based atlas will be used. The atlas is available online on [www.cancerdata.org](http://www.cancerdata.org) and will be updated whenever required.

An accurate delineation of the following structures is needed to deliver a safe irradiation and to assure delineation as similar as possible between the two arms.

- **Optic nerves:** delineated from the posterior edge of the eyeball, through the bony optic canal, where it narrows slightly, to the optic chiasm.
- **Lens**: a clearly visible biconvex avascular structure located between the vitreous humor and the iris and can easily be delineated on CT.
- **Optic Chiasm:** A T1 weighted MR (axial, sagittal and coronal) is recommended for delineation of the optic chiasm.
- **Optic Band:** A T1 weighted MR (axial, sagittal and coronal) is recommended for delineation of the optic band
- **Pituitary:** Oval-shaped (craniocaudally up to 12 mm) and lies in the sella turcica. Laterally, the pituitary gland is bordered by the cavernous sinuses, which are well visible with intravenous contrast agent. The inner part of the sella turcica can be used as a surrogate anatomical bony structure best identified using bone 1500/950 or soft tissue 350/50 WL/WW on CT.
- **Hypothalamus:** The hypothalamus (2–4 cm^3^) is a polygonal structure consisting of two separate volumes on each side of the third ventricle, delineated using MR. The mammillary bodies should be included in the contour. The medial border consists of the third ventricle or the visible CSF space. Since the lateral border is not clearly visible, the contour is bounded laterally 3 mm from the third ventricle.
- **Cochlea:** Using a WW/WL setting of 120/1500 on CT images, its volume can be defined as a small cavity. The semicircular canals should not be included.
- **Vestibular and semicircular canal**: delineation is advised using the bone setting on CT images (WW/WL 120/1500). The semicircular canal is located laterally and cranially of the cochlea. Vestibular is included in the same volume.
- **Brainstem**: Separated in three volumes: the whole brainstem (included medulla oblongata, pons, and midbrain; These structures do not have to be delineated), the brainstem interior is the brainstem surface contour cropped by 2 mm (inner border) and the brainstem surface (= the brainstem excluding the brainstem interior). Upper limit: from the nigral substance at the cerebral peduncle. Lower limit: tip of the dens of C2
- **Hippocampi**: The hippocampus (HC) is delineated as the gray matter medial to the medial boundary of the temporal horn of the lateral ventricle, bordered medially by the quadrigeminal cistern.
- **Lacrimal glands**: The lacrimal gland is an almond shaped gland (18 mm craniocaudally, 15 mm axial length and 5 mm axial width) located in the orbit superior-lateral to the eye. It can be delineated on CT using soft brain 120/40 or soft tissue 350/50 WW/WL settings.
- **Brain:** The delineation of the brain includes the cerebellum, CSF and small brain vessels, and excludes the brainstem and large cerebellar vessels, such as the sigmoid sinus, transverse sinus and superior sagittal sinus. In the middle cranial fossa the carotid canal and cavernous sinuses, most easily seen on contrast-enhanced T1 MRI, should not be included
- **Retina:** The retina is a neurosensorial membrane of 2mm thickness. Using a 2 mm inner wall, it can be delineated on CT as a membrane covering the posterior 5/6 of the globe, extending nearly as far as the ciliary body.
- **Cornea:** located at the anterior segment of the eyeball. It can be delineated as a 2mm inner wall. Cornea can easily be delineated on CT.
- **Eye ball**

### Dosimetric contraints

**For target coverage :**

- **Cohort A and C**
  - D98% >95% for the PTV or CTV if robust optimization is used
  - D95% > 98% for the PTV or CTV if robust optimization is used
  - D2% < 107% for te PTV or CTV if robust optimization is used
- **Cohort B**
  - D98%>100% for the PTV (Minor deviation if D98%>98%)
  - D2% >125% and < 140% for the PTV

**For organ at risk,** the following constraints should be respected:

| **COHORT B** | |
| --- | --- |
| **OAR** | **CONSTRAINTS** |
| Optic_nerve | D0,2cc <23Gy, Dmax <25Gy |
| Optic_Chiasm | D0,2cc <23Gy, Dmax <25Gy |
| Cochlea | Dmax<25Gy |
| Brainstem | D0,5cc <23Gy, Dmax< 31Gy |
| Encephalus-GTV | V30Gy<10,5cc |
| Optic_band | D0,2cc <23Gy, Dmax <25Gy |
| Cornea | As low as possible |
| Retina | D0,2cc <23Gy, Dmax <25Gy |
| HippocampiG+D | D40%<7,3Gy(EQD2) |
| Eye | Dmax<25Gy |
| Spinal_Cord | D0,35cc<23Gy , Dmax<30Gy |
| Lens | Dmax<3Gy |

| **COHORT A & C** | |
| --- | --- |
| **OAR** | **CONSTRAINTS** |
| Optic_nerve | Dmax<54Gy |
| Optic_Chiasm | Dmax<54Gy |
| Cochlea | Dmean< 45Gy / Dmean<32Gy |
| Brainstem | Dmax<60Gy, V54Gy< 33% |
| Encephalus-GTV | V50Gy<66%, V60Gy<33%, V45Gy<100% |
| Optic_band | Dmax<54Gy |
| Cornea | Dmax<50Gy |
| Retina | Dmax<45Gy |
| HippocampiG+D | D40%<7,3Gy(EQD2) |
| Eye | Dmax<45Gy, V30Gy<50% |
| Spinal_Cord | Dmax<45Gy |
| Lens | Dmax<10Gy |

In case of serial organs such as optic nerve priority is given to OAR. On the opposite, in case of parallel organs such as hippocampi, priority is given to PTV coverage.
